# Artificial Intelligence-Driven Multimodal Sensor Fusion for Complex Market Systems via Federated Transformer-Based Learning

**DOI:** 10.3390/s26082418

**Published:** 2026-04-15

**Authors:** Lei Shi, Mingran Tian, Yinfei Yi, Xinyi Hu, Xiaoya Wang, Yating Yang, Manzhou Li

**Affiliations:** 1 China Agricultural University, Beijing 100083, China; 2 National School of Development, Peking University, Beijing 100871, China

**Keywords:** artificial intelligence-driven sensing, multimodal sensor data fusion, cross-modal transformer, intelligent sensing systems, data-driven system analysis

## Abstract

In highly digitalized and networked modern trading systems, large volumes of heterogeneous data are continuously generated from multiple sources during market operations. However, due to the complexity of data structures, significant differences in temporal scales, and constraints imposed by data privacy protection, traditional single-source modeling approaches are unable to fully exploit multisource information. To address this issue, a federated multimodal prediction framework for complex market systems, termed Federated Market-Sensor Transformer (FMST), is proposed. In this framework, data originating from different information sources are uniformly modeled as multimodal time series. A multimodal market-sensor representation module is constructed to perform unified feature encoding, and a cross-modal Transformer fusion architecture is employed to characterize dynamic interaction relationships among different information sources. Meanwhile, a federated collaborative learning mechanism is introduced during the training phase, enabling multiple data nodes to perform collaborative model optimization without sharing raw data. In this manner, data privacy can be preserved while improving the cross-region generalization capability of the model. Systematic experimental evaluation is conducted on the constructed multimodal market-sensor dataset. The experimental results demonstrate that the proposed method consistently outperforms traditional statistical models and deep learning approaches across multiple evaluation metrics. In the main prediction experiment, FMST achieves a root mean square error (RMSE) of 0.1136, a mean absolute error (MAE) of 0.0832, and a coefficient of determination $R^{2}$ of 0.8517, while the direction prediction accuracy reaches 74.56%, clearly outperforming baseline models including ARIMA, LSTM, Temporal CNN, Transformer, and FedAvg-LSTM. In the cross-region generalization experiment, FMST maintains strong performance, achieving an RMSE of 0.1242, an MAE of 0.0908, an $R^{2}$ value of 0.8261, and a direction prediction accuracy of 72.48%. The ablation study further indicates that the three core components—multimodal market-sensor representation, cross-modal Transformer fusion, and federated collaborative learning—each make important contributions to the overall model performance. These experimental findings demonstrate that the proposed method can effectively integrate multisource market information and significantly enhance the prediction capability for complex market dynamics, providing a new technical pathway for the application of artificial intelligence-driven multimodal sensing systems in economic data analysis.

## 1. Introduction

With the rapid development of information technology and electronic trading systems, financial markets have entered a highly digitalized and networked stage [1]. From the perspective of the Adaptive Market Hypothesis, market systems are not static but evolve as participants continuously adapt their behaviors to changing environmental conditions and information landscapes [2]. This evolutionary process manifests through massive volumes of heterogeneous data across multiple sources, ranging from conventional price-volume sequences to granular order book depth, market sentiment, and macroeconomic indicators [3]. Each of these data modalities provides a distinct window into the underlying market state, yet the inherent information friction between distributed institutions often prevents a holistic understanding of market dynamics under traditional centralized paradigms [4]. In practical decision-making, participants must reconcile these diverse and fragmented signals to assess trends and risks in an increasingly interconnected global environment [5]. Consequently, the extraction and fusion of effective information from complex, multi-source data streams has become a critical research issue that bridges intelligent computing and financial economics [6]. The construction of analytical models capable of integrating these disparate data sources while respecting the distributed nature of market information is of considerable theoretical value for characterizing the dynamic variations of trading systems and promoting market stability [7].

Traditional financial forecasting has largely relied on classical statistical and econometric models—such as ARIMA [8], vector autoregression, GARCH [9], and regression based on technical indicators—which model market behavior [10] through linear relationships in historical data and have been widely applied in risk management and asset pricing [11]; however, these approaches typically assume linearity and stationarity and often depend on single data sources [12], limiting their ability to capture the nonlinear, non-stationary [13], and heterogeneous nature of real financial markets or integrate diverse information such as order book structures, news, and social media signals [14]. With advances in artificial intelligence, deep learning models—such as recurrent neural networks (RNNs), long short-term memory (LSTM), gate recurrent unit (GRU), convolutional neural networks (CNNs), and more recently, Transformer architectures—have increasingly been applied to financial forecasting [15], as their multilayer nonlinear structures and attention mechanisms enable automatic feature learning [16], effective modeling of long-range temporal dependencies, and natural fusion of multimodal data (e.g., price series, order book data, textual information, and macroeconomic indicators) [17], thereby improving the representation of complex market dynamics and predictive performance [18].

However, in real financial application environments, multi-source data are often distributed across different financial institutions or trading platforms [19], and these data are typically constrained by privacy protection, commercial confidentiality, and regulatory requirements, making direct sharing or centralized storage difficult [20]. Furthermore, significant differences exist across regional markets in terms of trading behavior, investment structure, and market mechanisms, which often lead to pronounced non-independent and identically distributed (non-IID) characteristics across data sources [21]. This phenomenon further increases the difficulty of model training and affects model stability in cross-region applications. Despite the advancements in federated learning for financial sentiment or risk control [22,23], a critical research gap persists: existing frameworks are not optimized to handle the extreme heterogeneity and multi-scale dynamics of market-sensor data—such as the interplay between high-frequency order books and sparse news events—under a unified federated architecture that specifically addresses cross-market distribution shifts. Most current methods lack the specialized mechanisms to adaptively synchronize these disparate signals or mitigate the impact of divergent trading mechanisms across global exchanges.

To bridge these gaps, this study proposes a federated multimodal financial data prediction framework, namely federated market-sensor transformer (FMST). Information from different data sources is represented as multimodal time-series data, and deep learning models are employed for feature extraction and fusion modeling. A federated learning mechanism is introduced to enable collaborative training across distributed nodes without sharing raw data. The main contributions of this study are summarized as follows.

1. We propose a multi-scale market-sensor representation method that incorporates a mixture-of-experts mechanism to adaptively encode price sequences, order book structures, textual news, and macroeconomic indicators, capturing market states from a holistic perspective.
2. We construct a cross-modal Transformer fusion module designed to model the intricate and dynamic dependencies across diverse market sensors, effectively enhancing the capture of market regimes and information shocks.
3. We design a robust federated optimization strategy using adversarial alignment and similarity distillation to mitigate the impact of non-IID characteristics across regional nodes, ensuring stable performance in heterogeneous market environments.
4. We validate the proposed framework on an extensive, real-world multi-source financial dataset, demonstrating superior performance in prediction accuracy and cross-region generalization compared to state-of-the-art baseline models [24].

## 2. Related Work

### 2.1. Deep Learning for Time-Series Sensing

Time-series data are widely encountered in numerous domains, including financial markets, industrial monitoring, and intelligent sensing systems, where the core characteristic lies in continuous temporal evolution accompanied by complex dynamic dependencies [25]. In early studies, time-series modeling was primarily dominated by statistical methods. However, with the continuous increase in computational capability and data scale, deep learning has gradually become an important technical paradigm for complex time-series modeling [26]. Through multilayer nonlinear architectures, deep learning models are capable of automatically learning latent features embedded in data, thereby demonstrating clear advantages in capturing complex dynamic patterns [27]. Among deep learning methods, RNNs represent one of the earliest important models applied to time-series modeling [28]. By introducing recurrent connections into the network structure, RNNs enable historical information to be utilized for predicting the current state and therefore are theoretically equipped with the capability to model sequential dependencies [29]. However, in practical training processes, conventional RNNs are prone to gradient vanishing and gradient explosion, which weakens their ability to learn long-term dependencies [30]. To address this issue, improved architectures such as LSTM and GRU were subsequently developed [31]. Through gating mechanisms including the input gate, forget gate, and output gate, LSTM effectively regulates the flow of information within the network, thereby strengthening the ability to capture long-term dependencies [32]. GRU adopts a more simplified gating structure and reduces model complexity while preserving sequence modeling capability; consequently, it has been widely applied in many time-series forecasting tasks [33]. In addition to RNNs, CNNs have also been gradually introduced into time-series analysis [34]. Unlike conventional RNNs, CNNs extract local temporal pattern features by sliding convolution kernels along the temporal dimension, and this strategy is effective in capturing short-term fluctuation patterns [35]. Convolution-based time-series models, such as Temporal CNN and temporal convolutional network (TCN), further enlarge the receptive field by incorporating dilated convolutions and residual structures, thereby enabling long-range dependency modeling to a certain extent while maintaining computational efficiency [36]. In recent years, with the development of attention mechanisms, the Transformer architecture has gradually become an important approach for time-series modeling [37]. Through the self-attention mechanism, correlations between any two time points in a sequence can be directly computed, thereby allowing long-range dependencies to be captured more flexibly. Compared with conventional recurrent structures, Transformer does not rely on sequential computation order and therefore provides stronger parallelization capability, which is favorable for efficient training under large-scale data settings [38]. In addition, the self-attention mechanism can dynamically adjust the importance weights of different time steps according to data content, thereby improving the identification of critical features [39]. In complex system modeling, Transformer can further achieve multi-scale feature learning through multi-head attention, which is particularly important for handling high-frequency dynamic data. In financial market forecasting research, deep learning methods for time-series analysis have been widely applied to tasks such as price prediction, volatility forecasting, and trading behavior analysis [40].

### 2.2. Multimodal Sensor Data Fusion

Multimodal data refer to data types that differ in source, structure, or representational form, such as structured numerical data, textual data, image data, and signal data [41]. Different modalities usually contain complementary information, and model understanding of complex systems can be significantly enhanced through appropriate fusion strategies [42]. In financial market research, multimodal data fusion mainly involves multiple information sources, including price sequences, order book structures, news text, and macroeconomic indicators. These data reflect market changes from different levels and are of substantial importance for improving market prediction capability [43]. The core issue of multimodal data fusion lies in how data of different types can be mapped into a unified feature space and subsequently integrated effectively [44]. Traditional approaches usually adopt feature concatenation for fusion, where feature vectors from different modalities are directly connected and then fed into a unified model for training [45]. Although this strategy is simple to implement, it is often difficult in practice to fully exploit the correlations among different modalities, and the resulting fusion effectiveness is therefore limited [46]. With the advancement of deep learning, multimodal fusion methods based on representation learning have been developed, in which latent relationships among different modalities are automatically learned by neural networks. For example, some studies have employed independent encoders to extract features from different modalities separately and then achieved feature alignment through a shared latent space, thereby constructing a unified representation [47]. In recent years, attention mechanisms have been widely applied in multimodal data fusion [48]. Transformer-based multimodal models further extend this idea by using multi-head attention structures to achieve interactive learning among multiple information sources, thereby exhibiting stronger feature representation capability under complex data environments [49]. In practical financial applications, multimodal data fusion can not only improve predictive performance but also enhance the interpretability of models with respect to market dynamics [50]. However, significant differences often exist across modalities in temporal frequency, data scale, and distribution characteristics, which increases the difficulty of data processing and model training [51]. Therefore, the construction of fusion models capable of adapting to complex multimodal data structures has become an important research direction in intelligent analytics for multi-source data [52].

### 2.3. Federated Learning for Distributed Sensing Systems

With the continuous growth of data scale and the increasing demand for data privacy protection, traditional centralized training paradigms have gradually encountered new challenges [53]. Under centralized training frameworks, all data are usually collected in a single server or data center for model training. Although this strategy can theoretically maximize the utilization of data resources, it is often constrained in practical applications by privacy protection requirements, data security concerns, and regulatory policies [54]. Particularly in sensitive domains such as finance, healthcare, and industrial data, raw data are often difficult to share directly across institutions, which imposes substantial obstacles on the practical deployment of traditional centralized machine learning methods [55]. Federated learning is a novel distributed machine learning framework whose fundamental idea is to achieve collaborative training across multiple nodes without sharing raw data [56]. Within the federated learning paradigm, each participating node trains a model locally using its own data and uploads model parameters or gradient information to a central server for aggregation [57]. After parameter aggregation is completed, the updated global model is redistributed to all participating nodes, thereby enabling continuous model optimization [58]. Through this mechanism, federated learning can make full use of distributed data resources while preserving data privacy, and it has therefore attracted broad attention in recent years [59]. However, in distributed sensing systems or cross-institution data environments, federated learning still faces several challenges [60]. First, data distributions across different nodes often differ substantially, leading to the non-independent and identically distributed (non-IID) problem, which can destabilize training and degrade global model performance [61]. Second, disparities in data scale and computational capability across nodes may affect the efficiency of model updates [62]. In addition, under multimodal data environments, the data types available at different nodes may not be fully consistent, which further increases the complexity of model design [63]. Therefore, how multimodal data can be collaboratively modeled within a federated learning framework while improving stability and generalization under heterogeneous data environments has become an important research topic [23].

### 2.4. Comparative Analysis of Federated Multimodal Learning

The rapid evolution of distributed sensing has led to various federated and multimodal learning paradigms, yet existing methods often exhibit limitations when applied to the high-frequency and heterogeneous environment of complex market systems. Traditional federated approaches, such as FedAvg-LSTM [64], primarily focus on homogeneous data structures and lack specialized mechanisms to capture the intricate interactions between disparate modalities like order book depth and news sentiment. While recent frameworks such as HA-Fedformer [24] have attempted to fuse knowledge across multimodal clients, they frequently assume synchronized data availability and often struggle with the severe non-IID problems caused by divergent regional trading mechanisms. To clarify the positioning of our work, Table 1 provides a systematic comparison between the proposed FMST framework and several representative state-of-the-art approaches.

As illustrated in the comparison, the FMST framework introduces several key improvements over prior studies. First, unlike conventional models that rely on simple feature concatenation [45], our approach utilizes a cross-modal Transformer that dynamically models dependencies across different temporal scales. Second, while existing federated methods often experience performance degradation under heterogeneous data distributions [61], FMST incorporates adversarial alignment and similarity distillation to specifically mitigate the impact of regional non-IID characteristics. Finally, the introduction of the mixture-of-experts (MoE) mechanism allows our framework to adaptively weigh the contribution of different market sensors, an essential feature for capturing information shocks that traditional fixed-weight fusion models often overlook. By addressing these specific limitations of prior research, FMST provides a more robust and adaptive solution for global financial market analysis.

## 3. Materials and Method

### 3.1. Data Collection

To construct a multimodal sensing dataset capable of reflecting the multi-source information structure of complex market systems, price trading data, order book depth data, textual information data, and macroeconomic indicator data were collected from multiple public data platforms and professional data interfaces. The data collection process involved automated ETL (Extract, Transform, Load) pipelines implemented using Python-based scheduling scripts, ensuring consistent data typology and integrity across different sources. These heterogeneous data sources were aligned and integrated under a unified temporal scale to form a consistent multimodal time-series dataset, as shown in Table 2.

Price and trading data were primarily obtained from high-frequency transaction data interfaces of the New York Stock Exchange (NYSE) and the NASDAQ market, combined with publicly available historical data from the Shanghai Stock Exchange (SSE) and the Shenzhen Stock Exchange (SZSE) in the Chinese A-share market. The data collection period spans from January 2018 to December 2024, with a temporal resolution of 1 min. The dataset includes fundamental trading indicators such as opening price, closing price, highest price, lowest price, trading volume, and transaction value. Approximately 1200 highly liquid stocks were covered, resulting in about $4.8\times 10^{8}$ high-frequency price records. To further characterize the microstructure of market dynamics, order book depth data were obtained through exchange Level-2 market data interfaces. These records contain the top ten levels of bid and ask prices along with their corresponding order volumes, with updates occurring at a temporal resolution of 10 s. Such data not only reflect instantaneous supply–demand relationships in the market but also capture variations in liquidity and structural characteristics of trading behavior. In total, approximately $1.6\times 10^{8}$ order book snapshots were collected in this study.

Textual information data were primarily sourced from multilingual financial news and public information platforms, including Refinitiv, Bloomberg, and the Chinese listed company disclosure platform CNINFO. Data collection for textual sources was executed through professional API polling and customized web scraping frameworks using Beautiful Soup and Scrapy (v2.15.0). News titles, full textual content, publication timestamps, and related topic tags were extracted and subsequently aligned with transaction data using timestamp synchronization. As a result, a textual dataset containing approximately $1.02\times 10^{6}$ English financial news articles and $7.8\times 10^{5}$ Chinese announcements and news reports was constructed. In addition, to capture the influence of macroeconomic environments on market behavior, macroeconomic indicators were collected from the World Bank database, the Federal Reserve Economic Data (FRED) system, and the public statistical database of the National Bureau of Statistics. The collected indicators include interest rates, inflation rates, industrial production indices, consumer confidence indices, and exchange rate indicators, resulting in approximately $2.4\times 10^{4}$ weekly and monthly macroeconomic records. All numerical data entered the system as structured float-point arrays, while textual data were initially stored as raw UTF-8 strings before being processed by semantic encoders. During the dataset construction process, all data sources were first synchronized along a unified temporal axis, after which a sliding-window mechanism was employed to generate multimodal time-series samples. Through this procedure, a comprehensive multimodal market-sensor dataset was established for subsequent model training and evaluation.

### 3.2. Data Preprocessing and Data Augmentation

In multi-source time-series modeling tasks, data originating from different sources often exhibit heterogeneous characteristics, including varying sampling frequencies, inconsistent data scales, and noise interference. If raw data are directly fed into deep learning models for training, unstable convergence may occur and the final prediction performance may be negatively affected. Therefore, systematic preprocessing is required prior to model training to improve data quality and enhance the ability of the model to learn complex dynamic patterns. During the construction of the multimodal time-series dataset, several preprocessing procedures were conducted, including temporal synchronization, outlier filtering, feature normalization, and sliding-window sampling. On this basis, a temporal perturbation augmentation strategy was further introduced to improve the robustness of the model with respect to time-series variations.

First, different data sources usually differ in sampling frequency and time-recording formats; therefore, temporal synchronization is required to align multi-source data onto a unified time scale. To properly account for cross-regional discrepancies, all raw timestamps from different global exchanges and news platforms are uniformly converted to Coordinated Universal Time (UTC). Let the raw time series from the *k*-th data source be denoted as $\left\{x_{t}^{\left(k\right)}\right\}$, where *t* represents the time index. Since the sampling timestamps across different sources are not completely consistent, they must be mapped to a unified temporal grid $\left\{\tau_{1},\tau_{2},\ldots ,\tau_{T}\right\}$. In practical implementation, linear interpolation or resampling methods are commonly adopted to achieve temporal alignment. For instance, when a timestamp $\tau_{i}$ does not exist in the original sequence, the corresponding value can be obtained through linear interpolation between two adjacent timestamps:

(1) $$x_{\tau_{i}}^{\left(k\right)}=x_{t_{1}}^{\left(k\right)}+\frac{\tau_{i}-t_{1}}{t_{2}-t_{1}}\left(x_{t_{2}}^{\left(k\right)}-x_{t_{1}}^{\left(k\right)}\right),$$

where $t_{1}$ and $t_{2}$ denote adjacent sampling timestamps satisfying $t_{1}<\tau_{i}<t_{2}$. In instances where information such as news or announcements is released during non-overlapping trading hours or market closures, the data are aggregated and mapped to the earliest available trading interval (market open) in the corresponding region. This procedure ensures that overnight information shocks or external market signals are appropriately integrated into the predictive features for the next trading session. Through this process, time series from different data sources are mapped to a unified temporal scale, forming an aligned multimodal time-series data matrix $X\in R^{T\times d}$.

After temporal synchronization, outlier handling is required. During high-frequency data acquisition, extreme values or noisy observations may occur due to device errors, network latency, or abnormal trading activities. Common outlier detection approaches include statistical distribution-based methods, such as thresholding based on mean μ and standard deviation σ. If an observation satisfies $|x_{t}-\mu |>\lambda \sigma$, it is regarded as an outlier, where λ is a threshold parameter typically set to 2 or 3. For detected outliers, we adopt a replacement strategy such as neighborhood averaging, where an outlier is replaced by the mean of its adjacent values, $x_{t}=\left(x_{t-1}+x_{t+1}\right)/2$. Through these procedures, the influence of noisy observations is effectively reduced.

After outlier processing, feature normalization is performed using a standardization strategy to map different features to a unified numerical scale. Given the original feature $x_{t}$ with mean μ and standard deviation σ, the standardized feature value is calculated as ${\tilde{x}}_{t}=\left(x_{t}-\mu \right)/\sigma$. This transformation ensures all features follow a standardized distribution with mean 0 and variance 1, thereby reducing the influence of feature scale differences and improving the convergence speed of the model.

Continuous time-series data are then converted into samples using the sliding-window technique. This method segments a long sequence into multiple subsequences $X_{i}=\left[x_{i},x_{i+1},\ldots ,x_{i+L-1}\right]$ by defining a fixed-length window *L*, with the corresponding prediction target defined as the future value $y_{i}=x_{i+L}$. By continuously sliding the window, a large number of training samples are constructed, preserving the temporal structure while enlarging the training sample size under limited data conditions.

To further improve adaptability, a temporal perturbation augmentation strategy is introduced. The main idea is to generate new training samples by random temporal shifts, $x_{t}^{\prime }=x_{t+\Delta t}$, where Δt is a random offset. Additionally, random noise $\epsilon_{t}∼N\left(0,\sigma^{2}\right)$ is added such that $x_{t}^{\prime }=x_{t}+\epsilon_{t}$. These perturbations expose the model to more diverse distributions, improving robustness against noise and temporal fluctuations.

### 3.3. Proposed Method

#### 3.3.1. Overall Formalization and Architecture Design

The proposed FMST framework is formalized as a multi-stage mapping function *F* that transforms heterogeneous input signals $X=\{X^{\left(p\right)},X^{\left(o\right)},X^{\left(t\right)},X^{\left(m\right)}\}$ into a predicted market state *Y*. Unlike traditional statistical models such as ARIMA [65], which rely on the assumption of linear stationarity and a limited number of variables, FMST is designed as a non-linear approximation model capable of handling high-dimensional and non-synchronous sensors. The first stage involves unified representation, where each modality *m* is mapped to a latent embedding $e^{\left(m\right)}=f_{m}\left(X^{\left(m\right)};\phi_{m}\right)$ using specialized encoders for price-volume sequences, order book structures, textual semantics, and macroeconomic context. This transformation ensures that disparate information sources are projected into a commensurate vector space $R^{d}$. The second stage utilizes a cross-modal Transformer architecture to model the joint distribution of these embeddings. While standalone LSTM architectures [66] process information sequentially and often struggle with long-range dependencies and modality interactions, FMST employs a multi-head attention mechanism $Attention\left(Q,K,V\right)=\mathrm{softmax}\left(\frac{QK^{⊤}}{\sqrt{d_{k}}}\right)V$. This allows the model to compute an interaction matrix where the importance of news sentiment or order book liquidity is adaptively weighted relative to price movements. This architectural choice addresses the limitations of Temporal CNNs [67] by offering a global receptive field over both temporal and modality dimensions. The final stage is the federated collaborative optimization, which solves a distributed objective $\min_{\theta }\sum_{i=1}^{N}\omega_{i}L_{i}\left(\theta \right)$, where $\omega_{i}$ is the relative weight of the *i*-th regional node. This stage differentiates FMST from centralized deep learning approaches by enabling global knowledge integration without compromising data privacy. By combining these formal components, the FMST framework establishes a rigorous mathematical pipeline that integrates local multimodal perception with global distributed optimization.

#### 3.3.2. Multimodal Market-Sensor Representation Module

In the multimodal market-sensor representation module, aligned feature sequences from different information sources are first received and treated as input signals from multiple sensing channels.

**Figure 1:**
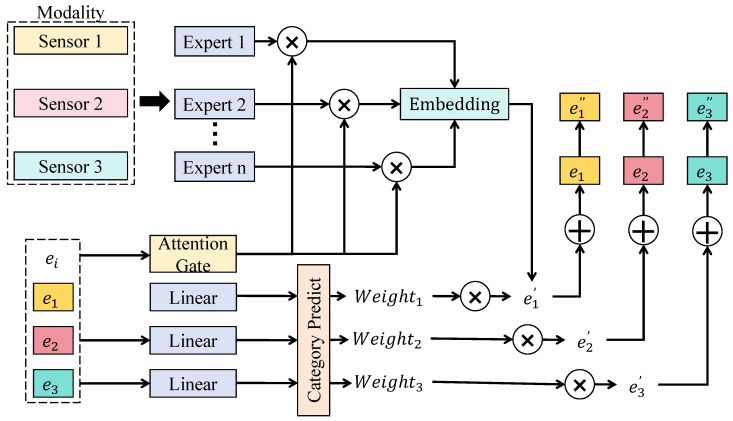
Architecture of the multimodal market-sensor representation module.

As shown in Figure 1, assume that within a temporal window *t*, there exist *M* modality inputs, where each modality corresponds to a feature sequence $X^{\left(m\right)}=\left\{x_{t-T+1}^{\left(m\right)},\ldots ,x_{t}^{\left(m\right)}\right\}$. In order to characterize the structural heterogeneity among modalities in the market system, an independent encoding branch is constructed for each modality, and a shared linear projection is applied to map all modalities into a unified representation space. For a modality *m*, a hidden representation is first obtained through the modality encoder as $h^{\left(m\right)}=f_{m}\left(X^{\left(m\right)}\right)$. This representation is then projected into a unified embedding through the projection layer as $e^{\left(m\right)}=W_{m}h^{\left(m\right)}+b_{m}$, where $W_{m}$ denotes the modality mapping matrix. Through this transformation process, price sequences, order book structures, textual semantics, and macroeconomic indicators are all converted into market sensing vectors with a unified dimensionality.

The incorporation of a mixture-of-experts (MoE) structure in the unified embedding space is motivated by the inherent regime-switching nature of financial markets. Traditional single-network models often struggle to generalize across disparate market states, such as high-volatility crises and low-volatility stable periods. By employing an MoE architecture, the framework can dedicate specialized expert networks to capture distinct market patterns or regimes, thereby enhancing the model capacity to approximate complex, non-linear market functions. Assume that the system contains *K* expert networks, each associated with a nonlinear transformation function $g_{k}\left(\cdot \right)$ that captures different types of market patterns. For an input embedding *e*, the output of each expert is given as $z_{k}=g_{k}\left(e\right)$. The contributions of different experts are dynamically assigned through an attention-based gating mechanism. This gating mechanism is selected over static weighting to allow the model to perform soft selection of experts based on the current contextual state of the market. The gating weight can be written as

(2) $$\alpha_{k}=\frac{\exp(a_{k}^{⊤}e)}{\sum_{j=1}^{K}\exp\left(a_{j}^{⊤}e\right)},$$

where $a_{k}$ represents the gating parameter vector. The final expert-fused representation is then obtained as

(3) $$\hat{e}=\sum_{k=1}^{K}\alpha_{k}z_{k}.$$

Such a mixture structure allows adaptive selection of transformation pathways under different market environments, thereby improving the adaptability of the model to heterogeneous market structures.

After obtaining the expert-fused representation, attention-based gating is further applied to integrate different modality signals. The choice of attention-based gating for modality fusion is driven by the dynamic information value of different sources; for instance, news sentiment may become the dominant predictor during earnings seasons, while order book depth is more critical during periods of high-frequency trading. Static fusion methods fail to capture these temporal shifts in information importance. Assume that modality embeddings are denoted as $e_{p}$, $e_{o}$, $e_{t}$, and $e_{m}$, corresponding to price, order book, text, and macroeconomic information. The gating network generates modality weights through a scoring function

(4) $$w^{\left(m\right)}=\frac{\exp(u^{\left(m\right)})}{\sum_{j}\exp\left(u^{\left(j\right)}\right)},$$

where $u^{\left(m\right)}$ denotes the score produced by the gating network. The multimodal fusion representation is therefore expressed as

(5) $$e_{fusion}=\sum_{m=1}^{M}w^{\left(m\right)}e^{\left(m\right)}.$$

This representation captures the relative contribution of different information sources to the current market state.

To further enhance task relevance, a feature modulation mechanism is introduced on the fused representation. The vector $e_{fusion}$ is first passed through a prediction network to generate intermediate weights $w_{d}$, $w_{t}$, and $w_{v}$. These weights are applied to modulate modality features, producing modulated representations such as

(6) $${\tilde{e}}^{\left(m\right)}=w^{\left(m\right)}⊙e^{\left(m\right)},$$

where ⊙ denotes element-wise multiplication. The final representation is obtained through residual aggregation

(7) $$e^{*}=e_{fusion}+\sum_{m}{\tilde{e}}^{\left(m\right)}.$$

This feature modulation and residual aggregation design ensures that the model can highlight task-specific salient features while maintaining a robust gradient flow through the network. Through these designs, the multimodal representation module enables deep integration of heterogeneous information sources in a unified space, dynamically selecting the most informative feature expressions via mixture-of-experts and attention gating, which enhances the representation of complex market dynamics and provides high-quality inputs for subsequent cross-modal fusion.

#### 3.3.3. Cross-Modal Sensor Fusion Transformer

After obtaining the unified embedding vectors from the multimodal market-sensor representation module, the cross-modal Transformer fusion module performs deep modeling of dynamic dependencies among different modalities.

**Figure 2:**
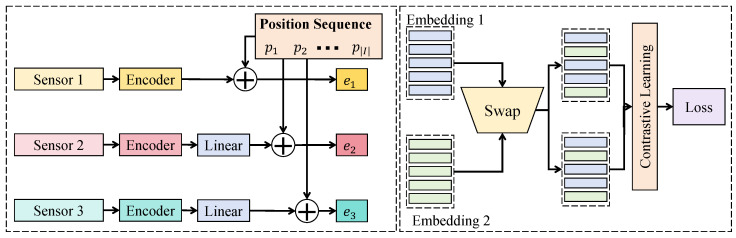
Architecture of the cross-modal sensor fusion transformer.

As shown in Figure 2, assume that the multimodal representations within a temporal window form a sequence $E=\{e_{1},e_{2},\ldots ,e_{T}\}$, where each $e_{t}\in R^{C}$ represents the comprehensive market sensing vector at time *t*. The input sequence is first projected through linear transformations to construct attention representations. The feature width corresponds to *C*, while the sequence height corresponds to *T*, and the representation is internally divided into multiple channel subspaces to enhance representation capacity. The fusion network is composed of stacked Transformer encoder blocks, where each layer contains a cross-modal attention sublayer and a feed-forward sublayer, connected through residual pathways and layer normalization. For the input representation of the *l*-th layer $H^{\left(l\right)}$, the cross-modal attention representation is computed as

(8) $$A^{\left(l\right)}=\mathrm{softmax}\left(\frac{H^{\left(l\right)}W_{q}^{\left(l\right)}{\left(H^{\left(l\right)}W_{k}^{\left(l\right)}\right)}^{⊤}}{\sqrt{d}}\right)H^{\left(l\right)}W_{v}^{\left(l\right)},$$

where $W_{q}^{\left(l\right)},W_{k}^{\left(l\right)},W_{v}^{\left(l\right)}$ denote learnable parameter matrices. This structure models correlations across both temporal positions and modality features through the attention weight matrix. Multiple attention heads are then employed to learn representations across different feature subspaces

(9) $$\mathrm{MHA}\left(H^{\left(l\right)}\right)=\mathrm{Concat}\left(A_{1}^{\left(l\right)},A_{2}^{\left(l\right)},\ldots ,A_{h}^{\left(l\right)}\right)W_{o}^{\left(l\right)},$$

where each attention head $A_{i}^{\left(l\right)}$ captures interactions in a different channel subspace. This design enables the model to simultaneously learn information interactions across different scales. After attention fusion, nonlinear feature transformation is applied through a position-wise feed-forward network

(10) $$F^{\left(l\right)}=\sigma \left(H^{\left(l\right)}W_{1}^{\left(l\right)}+b_{1}^{\left(l\right)}\right)W_{2}^{\left(l\right)}+b_{2}^{\left(l\right)},$$

where σ(·) denotes the nonlinear activation function. The output of the layer is then obtained through residual integration

(11) $$H^{\left(l+1\right)}=\mathrm{LayerNorm}\left(H^{\left(l\right)}+\mathrm{MHA}\left(H^{\left(l\right)}\right)+F^{\left(l\right)}\right).$$

Stacking multiple layers allows the network to gradually construct higher-level cross-modal semantic structures. To strengthen the interaction among modalities, modality positional encoding and temporal positional encoding are added to the input sequence

(12) $$\tilde{E}=E+P_{time}+P_{modal}.$$

This design allows the model to capture both temporal order and modality identity simultaneously. Theoretically, such a structure can approximate arbitrary cross-modal interaction functions in the feature space. Let the fusion mapping be denoted as f(E), which under the multi-head attention structure can be written as

(13) $$f\left(E\right)=\sum_{i=1}^{h}\alpha_{i}\left(E\right)\phi_{i}\left(E\right),$$

where $\alpha_{i}\left(E\right)$ represents the attention weight function and $\phi_{i}\left(E\right)$ denotes the transformation function in the corresponding subspace. According to universal function approximation theory, when the number of attention heads and network depth are sufficiently large, the structure is capable of approximating complex feature interaction functions. Furthermore, since attention weights satisfy the normalization property

(14) $$\sum_{j}a_{ij}=1,$$

the fused representation can be interpreted as a weighted combination of modality features, ensuring stability and preventing feature explosion. In practical implementation, the cross-modal Transformer module adopts stacked layers that maintain temporal sequence height while expanding feature channels through multi-head mechanisms. This architecture jointly models temporal dependencies and modality interactions, enabling unified representation of the complex coupling among price dynamics, order book structure variations, news events, and macroeconomic conditions, thereby improving prediction robustness in complex market environments.

#### 3.3.4. Federated Collaborative Learning Mechanism

After cross-modal fusion, the unified market state sequence is fed into the federated collaborative learning mechanism to enable distributed training and knowledge integration.

**Figure 3:**
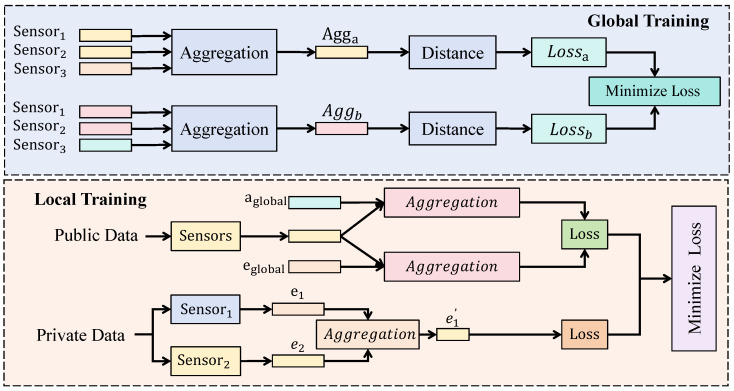
Architecture of the federated collaborative learning mechanism.

As shown in Figure 3, assume that the system contains *N* participating nodes, where each node possesses a local dataset $D_{i}$ and deploys a complete FMST network locally. Each node receives global model parameters from the central server and performs multiple rounds of gradient updates based on its local data. During local training, the fused representation is first passed through a prediction network composed of several fully connected layers that expand feature dimensions while maintaining a temporal structure. The local model parameter is denoted as $\theta_{i}$, and the training objective is expressed as

(15) $$L_{i}\left(\theta_{i}\right)=E_{\left(x,y\right)∼D_{i}}\;\ell \left(f_{\theta_{i}}\left(x\right),y\right),$$

where $f_{\theta_{i}}\left(x\right)$ represents the prediction function at node *i*.

To mitigate distribution discrepancies across nodes, an adversarial alignment mechanism is introduced during local training. The choice of adversarial alignment is specifically targeted at the non-IID (non-Independent and Identically Distributed) nature of global market data. Different regional exchanges often exhibit distinct trading frequencies and volatility patterns; by incorporating a discriminator to challenge the feature encoder, we force the model to extract domain-invariant representations. This ensures that the learned market features reflect universal economic laws rather than over-fitting to region-specific noise, thereby significantly enhancing cross-market generalization. Let $z_{i}$ denote the local feature representation and $d(z_{i})$ denote the output of a discriminator. The adversarial objective can be written as

(16) $$L_{adv}=E_{z_{i}}\log d\left(z_{i}\right)+E_{z_{j}}\log\left(1-d\left(z_{j}\right)\right).$$

This mechanism encourages feature distributions from different nodes to gradually align within the latent space, thereby reducing the influence of cross-region distribution shifts.

After local training, model parameters from all nodes are transmitted to the server for aggregation. The server constructs the global model by weighted parameter aggregation. Let the data size of node *i* be $|D_{i}|$, and the global update rule is

(17) $$\theta^{\left(t+1\right)}=\sum_{i=1}^{N}\omega_{i}\theta_{i}^{\left(t\right)},$$

where the weight coefficient satisfies

(18) $$\omega_{i}=\frac{|D_{i}|}{\sum_{j=1}^{N}\left|D_{j}\right|}.$$

This strategy ensures that each node contributes proportionally according to its data scale, improving stability and convergence efficiency.

To further enhance the generalization capability of the global model, a similarity-based distillation mechanism is introduced at the server side. This distillation mechanism is designed to facilitate high-order knowledge sharing between nodes without the exchange of raw data. By treating each local model as a specialized teacher, the global model can inherit fine-grained insights from diverse market environments. This prevents the global parameters from drifting due to conflicting local updates and ensures that the final representation remains consistent and stable across the entire federated network. Let $z_{i}$ and $z_{j}$ denote embeddings from nodes *i* and *j*. The distillation objective is expressed as

(19) $$L_{kd}={\left|z_{i}-z_{j}\right|}_{2}^{2}.$$

The server minimizes the distillation loss to encourage knowledge consistency across nodes, resulting in a more stable global representation. The overall training objective becomes

(20) $$L_{global}=\sum_{i=1}^{N}L_{i}+\lambda_{1}L_{adv}+\lambda_{2}L_{kd},$$

where $\lambda_{1}$ and $\lambda_{2}$ balance different loss components. Through this design, federated collaborative learning enables cross-node knowledge sharing while preserving data privacy, and the adversarial alignment and distillation mechanisms alleviate non-IID distribution issues, thereby improving stability and generalization in complex market prediction tasks.

## 4. Results and Discussion

### 4.1. Experimental Configuration

#### 4.1.1. Experimental Setting

The experiments were conducted on a high-performance server equipped with an NVIDIA A100 GPU (80 GB VRAM) and a 64-core AMD EPYC 7742 CPU with 256 GB DDR4 RAM. This hardware suite supports large-scale matrix operations and parallelized processing of multimodal time-series data. The software environment utilized Ubuntu 22.04 LTS as the operating system. Models were implemented using Python 3.10.12 and PyTorch 2.2.0. Data processing and evaluation metrics were managed with NumPy 1.26.4, Pandas 2.2.1, and Scikit-learn 1.4.1, while Matplotlib 3.7.2 was used for visualization.

The dataset was partitioned chronologically into training (70%), validation (15%), and test (15%) sets. The training set facilitated parameter learning, while the validation set was used for hyperparameter tuning and early stopping. To ensure results were robust, we implemented a 5-fold cross-validation strategy where the average performance across runs was reported as the final evaluation.

We performed a grid search to determine the optimal hyperparameters for the FMST framework. The search space included learning rate $\alpha \in \{1\times 10^{-4},5\times 10^{-4},1\times 10^{-3},5\times 10^{-3}\}$, batch size ∈{32,64,128}, and Transformer hidden dimension $d_{model}\in \left\{64,128,256\right\}$. The number of attention heads *h* was tested within {4,8,16}, and the feed-forward dimension $d_{ff}$ within {256,512,1024}. For time-series input, we evaluated sliding-window lengths L∈{15,30,45,60}.

The final configuration used the Adam optimizer with a learning rate of α=0.001 and a batch size of 64. Training was limited to 100 epochs with early stopping to prevent overfitting. Within the Transformer, we set h=8, $d_{model}=128$, and $d_{ff}=512$. The sliding-window length was fixed at L=30. In federated learning settings, the local training epochs *E* were set to 5 and global communication rounds *R* to 50, using a sample-size-weighted aggregation strategy.

#### 4.1.2. Baseline Models and Evaluation Metrics

In the comparative experiments, several representative baseline models were selected as references, including ARIMA [65], LSTM [66], Temporal CNN [67], Transformer [68], and FedAvg-LSTM [64], in order to comprehensively evaluate the performance of the proposed method. The specific configuration of input data for each method is summarized in Table 3.

First, ARIMA (AutoRegressive Integrated Moving Average) is a classical time-series forecasting model whose basic idea is to characterize the linear dependency structure of a sequence through autoregressive, differencing, and moving average terms. It is usually represented in the form of ARIMA(p,d,q), where p denotes the autoregressive order, d denotes the differencing order, and q denotes the moving average order. This model is limited to modeling univariate historical price data. Second, LSTM is a recurrent neural network architecture that utilizes gating mechanisms to regulate information flow and capture temporal dependencies. Temporal CNN extracts local dynamic features by applying convolution kernels along the temporal dimension. The Transformer model models long-term dependencies through the self-attention mechanism and facilitates interaction between different features. Finally, FedAvg-LSTM is a distributed learning method that aggregates LSTM parameters across nodes to achieve collaborative learning while preserving data privacy. To ensure a fair comparison, all deep learning baselines (LSTM, Temporal CNN, Transformer, and FedAvg-LSTM) are provided with the same multimodal input features as the proposed FMST framework.

To comprehensively evaluate model performance in the time-series prediction task, four evaluation metrics were adopted in this study, namely root mean square error (RMSE), mean absolute error (MAE), coefficient of determination ($R^{2}$), and direction accuracy (DA). Among them, RMSE is used to measure the overall magnitude of the error between predicted values and true values and is more sensitive to large errors. MAE is used to quantify the average absolute magnitude of prediction errors and can reflect the overall prediction bias of the model. $R^{2}$ is used to assess the ability of the model to explain variations in data trends. DA is used to measure the correctness of the predicted direction of time-series changes, thereby evaluating the ability of the model to identify trends. The mathematical expressions of the above evaluation metrics are given as follows:

(21) $$RMSE=\sqrt{\frac{1}{N}\sum_{i=1}^{N}{\left(y_{i}-{\hat{y}}_{i}\right)}^{2}},$$

(22) $$MAE=\frac{1}{N}\sum_{i=1}^{N}\left|y_{i}-{\hat{y}}_{i}\right|,$$

(23) $$R^{2}=1-\frac{\sum_{i=1}^{N}{\left(y_{i}-{\hat{y}}_{i}\right)}^{2}}{\sum_{i=1}^{N}{\left(y_{i}-\bar{y}\right)}^{2}},$$

(24) $$DA=\frac{1}{N}\sum_{i=1}^{N}I\left(\left(y_{i}-y_{i-1}\right)\left({\hat{y}}_{i}-{\hat{y}}_{i-1}\right)>0\right).$$

In the above equations, *N* denotes the number of test samples, $y_{i}$ denotes the true value at the *i*-th time step, ${\hat{y}}_{i}$ denotes the predicted value of the model at the corresponding time step, $\bar{y}$ denotes the mean of the true values, and I(·) denotes the indicator function, which takes the value 1 when the condition inside the parentheses is satisfied and 0 otherwise. In the computation of DA, $\left(y_{i}-y_{i-1}\right)$ denotes the direction of change in the true sequence between adjacent time steps, while $\left({\hat{y}}_{i}-{\hat{y}}_{i-1}\right)$ denotes the direction of change in the predicted sequence. When the signs of the two terms are identical, the direction prediction is regarded as correct.

### 4.2. Main Prediction Performance Comparison

The primary objective of this experiment is to systematically evaluate the overall performance of different models on the multimodal market-sensor prediction task and to verify the effectiveness of the proposed FMST framework in modeling complex market dynamics. Under a unified data partition setting, comparative analyses were conducted among traditional time-series models, deep learning models, and federated learning models. The federated learning system consists of eight collaborative clients, each corresponding to a regional market data source, and a global model is obtained through multiple rounds of parameter aggregation.

The results in Table 4 indicate clear performance differences among the evaluated models across all metrics. As a traditional statistical model, ARIMA exhibits the highest RMSE and MAE values among all methods, with a direction prediction accuracy of only 61.38%, indicating clear limitations when modeling complex nonlinear market structures. LSTM shows a noticeable improvement over ARIMA across all evaluation metrics, with RMSE decreasing to 0.1316 and direction prediction accuracy increasing to 66.91%, suggesting that recurrent neural networks are capable of capturing temporal dependencies in time-series data to a certain extent. Temporal CNN further improves predictive performance through convolutional structures that model local temporal patterns, demonstrating the advantage of convolutional networks in capturing short-term structural fluctuations. The Transformer model achieves further improvements due to the use of self-attention mechanisms that model global temporal dependencies, with RMSE and MAE decreasing further and $R^{2}$ increasing to 0.8269, while direction prediction accuracy reaches 70.84%. FedAvg-LSTM performs collaborative training across multiple nodes within a federated learning environment, achieving certain improvements compared with the standalone LSTM model, although its performance remains slightly lower than that of the Transformer model. The proposed FMST method achieves the best results across all metrics, with RMSE of 0.1136, MAE of 0.0832, $R^{2}$ reaching 0.8517, and direction prediction accuracy increasing to 74.56%, indicating that the proposed framework captures complex market dynamics more effectively.

From the perspective of model structural characteristics and mathematical modeling capability, the performance differences among these approaches mainly arise from their ability to model temporal dependencies and interactions among multiple information sources. ARIMA is essentially a linear autoregressive model whose predictive ability depends on fixed-order combinations of historical observations, making it insufficient for capturing nonlinear structures and complex dynamics commonly observed in financial markets. LSTM employs gating mechanisms to recursively update hidden states and can learn certain long-term dependencies; however, information propagation still follows a sequential path along the temporal dimension, which may lead to information attenuation over long horizons. Temporal CNN performs local feature extraction through convolution kernels along the temporal dimension, enabling effective capture of short-term fluctuation patterns, although its receptive field remains constrained by convolutional structures. Transformer models establish direct relationships across all temporal positions through attention mechanisms, theoretically allowing dependencies between arbitrary time steps to be modeled, which leads to stronger representation capability in complex time-series prediction tasks. FedAvg-LSTM introduces a federated learning mechanism that enables knowledge sharing across nodes, thereby alleviating local data distribution bias; however, its underlying recurrent architecture still limits its overall representation capacity. In contrast, FMST incorporates multimodal market-sensor representation mechanisms on top of the global dependency modeling capability of Transformer architectures and integrates a federated collaborative learning framework. As a result, both cross-modal information interactions and cross-regional market regularities can be learned simultaneously, leading to higher predictive accuracy and stronger generalization ability in complex market prediction tasks.

**Figure 4:**
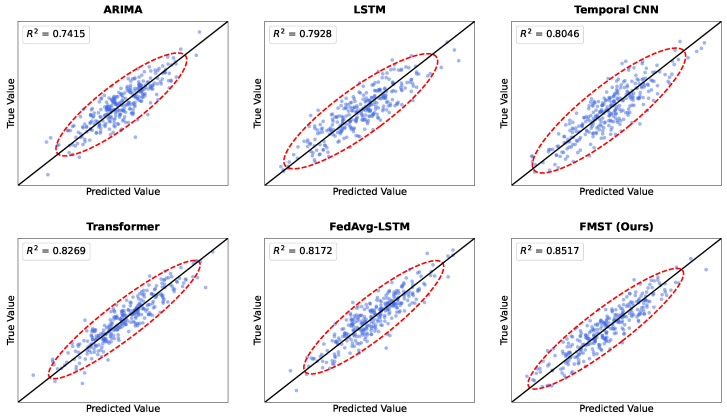
Comparison of predicted versus true values for different models on the multimodal market-sensor dataset.

The results illustrated in Figure 4 provide a visual comparison of the fitting performance among the evaluated models during the main prediction experiment. Each subplot displays the scatter distribution of predicted values against true values, with the diagonal line representing an ideal perfect prediction. It can be observed that the ARIMA model exhibits a relatively sparse and wide distribution of data points, which is consistent with its lower $R^{2}$ value of 0.7415 [65]. While deep learning baselines such as LSTM and Temporal CNN show improved clustering, our proposed FMST framework achieves the most concentrated alignment along the 45-degree diagonal [66,67]. The FMST scatter plot demonstrates a significantly tighter grouping with an $R^{2}$ of 0.8517, indicating that the integration of multimodal market sensors allows the model to capture the underlying market dynamics more accurately than single-source or simpler fusion architectures [64,68].

### 4.3. Cross-Region Generalization and Non-IID Impact Analysis

This experiment aims to evaluate the generalization capability of different models under cross-region market environments, namely whether a model trained in one regional market can be effectively transferred to another regional market for prediction. In real-world economic systems, different regions or trading markets often exhibit significant differences in investor structure, trading activity, and information dissemination mechanisms, leading to pronounced non-IID characteristics in the data. By constructing cross-region training and testing tasks, the robustness and adaptability of models under distribution shifts can be examined.

The experimental results in Table 5 show that the performance of all models decreases compared with the main experiment, indicating that distribution differences significantly affect prediction models. To provide a detailed analysis of the non-IID impact, we observe that the statistical heterogeneity across regional nodes—such as the disparity between price limit regulations in some Asian markets and the continuous volatility of Western markets—causes a phenomenon known as weight divergence during federated aggregation. When data are non-IID, the local optimal solutions for different nodes drift in opposite directions in the parameter space, leading to a suboptimal global model that struggles to generalize. Traditional models like ARIMA and standalone LSTM exhibit the most significant degradation because they lack mechanisms to separate region-specific noise from universal market laws. In contrast, the FMST method achieves the best performance with an RMSE of 0.1242 and DA of 72.48%. This superiority is attributed to the adversarial alignment and similarity distillation modules, which are designed to address the non-IID problem. By forcing the latent representations of different regional nodes into a domain-invariant feature space, the FMST framework effectively filters out the regional biases caused by varying trading mechanisms and investor behaviors. This ensures that the aggregated global model is grounded in common market dynamics rather than being skewed by local distributional outliers, thereby maintaining high stability even under significant cross-region shifts.

**Figure 5:**
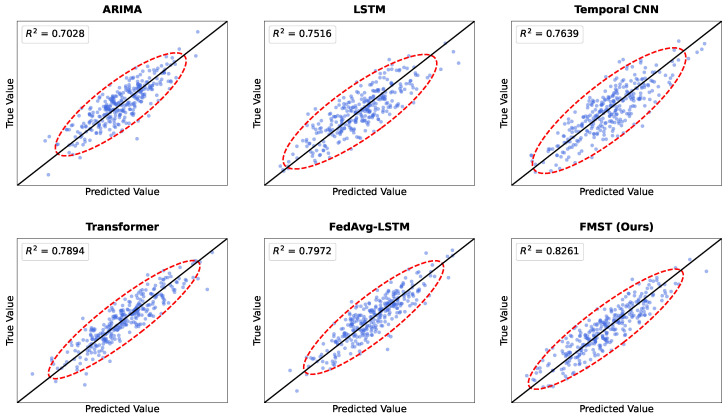
Comparison between predicted and true values of different models in the cross-region generalization experiment.

Figure 5 visualizes the model performance in the cross-region generalization experiment, where distribution shifts and non-IID characteristics pose significant challenges [21]. Compared to the main experiment, all models show a higher degree of dispersion in their scatter plots, reflecting the impact of regional market differences on predictive stability. The ARIMA and standalone LSTM models suffer from the most pronounced degradation, with points deviating substantially from the diagonal line [8]. In contrast, the FMST model maintains superior robustness, with the majority of its predictions remaining closely aligned with the true values, resulting in an R2 of 0.8261. This visual evidence supports our finding that the federated collaborative learning mechanism and cross-modal Transformer fusion effectively alleviate local distribution biases, enabling the model to learn universal market regularities that generalize well across diverse trading environments [64,68].

### 4.4. Ablation Study on the Core Module

This experiment aims to analyze the contribution of each core component in the proposed FMST framework to the overall model performance and to verify the effectiveness of different structural designs in complex market prediction tasks. By progressively removing the multimodal market-sensor representation module, the cross-modal Transformer fusion module, and the federated collaborative learning module, performance variations can be observed to evaluate the importance of each component.

**Figure 6:**
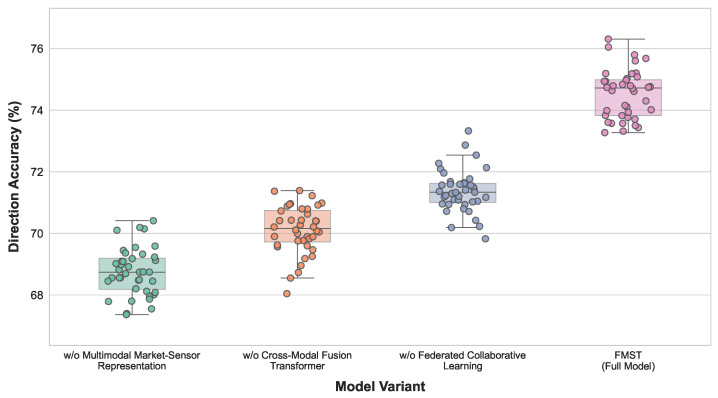
Distribution of direction prediction accuracy for different FMST model variants in the ablation experiment.

The experimental results in Table 6 and Figure 6 show that removing the multimodal market-sensor representation module leads to the most significant performance degradation, with RMSE increasing to 0.1267, MAE increasing to 0.0936, and direction prediction accuracy decreasing to 68.93%, indicating that this module plays a critical role in constructing unified market state representations. When the cross-modal Transformer fusion module is removed, the error metrics increase slightly, although the overall performance remains higher than the previous variant, with RMSE of 0.1239 and direction prediction accuracy of 70.14%. This observation indicates that cross-modal fusion is essential for capturing dynamic relationships among heterogeneous information sources. When the federated collaborative learning module is removed, the performance decreases again, with RMSE increasing to 0.1208 and direction prediction accuracy dropping to 71.36%, suggesting that federated learning contributes significantly to integrating cross-regional knowledge. The full FMST model achieves the best performance across all metrics, with RMSE of 0.1136, MAE of 0.0832, and direction prediction accuracy reaching 74.56%, demonstrating that the three modules form complementary components that jointly improve prediction performance.

From the perspective of model architecture and mathematical modeling capability, each module contributes to performance improvement through its role in feature representation, information fusion, and distribution learning. The multimodal market-sensor representation module encodes price sequences, order book structures, textual information, and macroeconomic indicators into a unified representation space, enabling comprehensive market state modeling. Without this module, the model receives only single-type features, which reduces representation capacity and limits the ability to capture complex interactions among heterogeneous information sources. The cross-modal Transformer fusion module establishes global dependencies among modalities through attention mechanisms, allowing the model to automatically learn the relative importance of different information sources. When this module is removed, the model relies on simple feature combinations, which reduces predictive accuracy. The federated collaborative learning module enables parameter sharing across multiple nodes, allowing the model to learn common structural patterns from different regional markets and thereby mitigating distribution bias from individual data sources. Without this module, training relies solely on local data, which weakens model stability and generalization capability. Overall, FMST integrates multimodal representation learning, cross-modal information fusion, and federated collaborative optimization, forming a synergistic architecture that improves both feature representation capacity and cross-environment adaptability, thereby achieving more accurate and robust market prediction results.

### 4.5. Ablation Study on Input Data Modalities

The primary objective of this experiment is to systematically evaluate the information contribution of different input data sources to the predictive performance of the FMST framework and to conduct a pragmatic cost–benefit analysis of these modalities. By isolating or progressively adding specific data modalities, including price-volume sequences, order book depth, financial news, and macroeconomic indicators, we aim to quantify the marginal benefit of heterogeneous signals in capturing complex market dynamics. This analysis provides empirical evidence regarding which information sources are most critical for achieving high-precision market forecasting and assesses whether the performance improvements justify the associated acquisition costs and computational requirements of the multimodal sensing approach adopted in this study.

As shown in Table 7, the experimental results demonstrate that while price and trading data serve as the fundamental basis for prediction, the integration of additional modalities yields significant and consistent performance gains. From a cost–benefit perspective, price data from public historical interfaces offer the highest information density relative to their low acquisition cost. The inclusion of order book depth information, obtained via exchange Level-2 APIs, enhances the model capability to capture micro-liquidity variations and supply–demand imbalances, leading to a noticeable reduction in root mean square error. While professional order book data incur higher subscription fees, the resulting 1.84 percent improvement in DA is substantial for high-frequency trading environments, where even marginal gains can lead to significant returns. Furthermore, the addition of textual news from premium sources like Bloomberg or Refinitiv provides critical sentiment signals that improve DA to 73.18 percent. Although news data are the most costly and time-consuming to process due to the need for semantic encoders and high-performance NLP pipelines, they prove indispensable for capturing event-driven shocks during periods of high volatility. The investment in these high-cost sources is justified by the enhanced risk mitigation and the model’s superior ability to identify structural market shifts. Macroeconomic indicators, which are largely accessible from public databases with minimal processing overhead, contribute to the overall stability of the model by providing essential contextual information. These findings confirm that the multimodal sensing strategy effectively leverages complementary information across different scales and structures to achieve superior robustness compared to single-source modeling approaches like ARIMA [8] or standalone LSTM [8].

### 4.6. Performance Comparison Under Different Numbers of Federated Clients

This experiment investigates the influence of the number of collaborative clients on prediction performance within the federated learning environment, thereby evaluating the effectiveness of federated collaborative training for complex market prediction tasks. Under consistent model architectures and training strategies, the number of participating clients is gradually increased to observe performance variations. Local training represents the scenario in which only a single node is used for model training without federated parameter aggregation, while the number of federated clients is progressively increased from two to eight to construct the global model through server-side aggregation.

**Figure 7:**
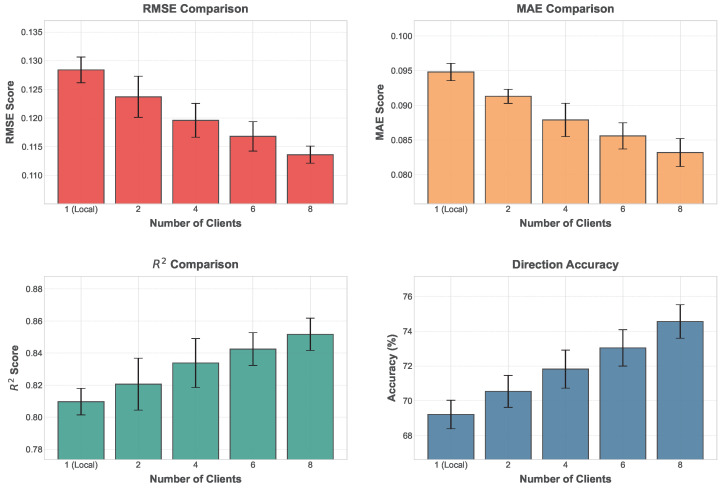
Performance comparison under different numbers of federated clients.

The results in Table 8 and Figure 7 demonstrate that prediction performance consistently improves as the number of participating federated clients increases. In the single-node training scenario, RMSE reaches 0.1284, MAE reaches 0.0948, and direction prediction accuracy is 69.21%, indicating that although certain market dynamics can be learned from a single data source, the predictive capability remains limited by data scale and distribution diversity. When the number of federated clients increases to two and four, noticeable improvements are observed across all evaluation metrics, with RMSE decreasing to 0.1237 and 0.1196 and direction prediction accuracy increasing to 70.54% and 71.82%, respectively. When the number of clients further increases to six and eight, prediction performance improves further. The best results are obtained when eight clients participate in collaborative training, where RMSE decreases to 0.1136, MAE decreases to 0.0832, and direction prediction accuracy reaches 74.56%, demonstrating that federated collaborative learning significantly enhances predictive capability.

From the perspective of model structure and mathematical modeling characteristics, the experimental results highlight the advantage of federated learning in integrating multi-source data. In the single-node training scenario, parameter updates are driven only by local market data, meaning that the learned feature patterns mainly reflect local market structures and may suffer from distribution bias when applied to complex market dynamics. As the number of federated clients increases, parameter updates from multiple nodes are aggregated during each training round, allowing shared structural patterns across different regional markets to be gradually learned. This collaborative updating mechanism forms more stable representations in parameter space and balances different data distributions. From a statistical perspective, federated aggregation effectively performs weighted integration of parameters learned from multiple local models, enabling the global model to incorporate statistical characteristics from diverse data sources and thereby reducing bias caused by single-source training. As the number of participating nodes increases, the model is exposed to richer market structural patterns and can learn more stable feature mappings. Consequently, both prediction error metrics and trend prediction accuracy improve consistently. These results confirm the effectiveness of federated collaborative learning in complex market prediction tasks and demonstrate that multi-node collaborative training significantly enhances model stability and generalization capability.

### 4.7. Parameter Sensitivity and Robustness Analysis

The primary objective of this experiment is to systematically verify the stability and robustness of the FMST framework under varying hyperparameter settings. By exploring a range of values for critical architectural parameters, such as the sliding-window length L and the number of attention heads h, we aim to identify the optimal configuration for complex market prediction. This analysis ensures that the proposed framework is not overly sensitive to specific initial conditions and can maintain consistent performance across diverse deployment scenarios, thereby providing empirical evidence for its practical reliability in real-world trading environments.

As shown in Table 9, the experimental results indicate that the FMST framework maintains a high degree of stability across various configurations. Regarding the sliding-window length L, performance improves as L increases from 15 to 30, suggesting that capturing a sufficient historical context is essential for modeling short-term temporal dynamics. However, further increasing L to 60 leads to a slight decrease in accuracy and an increase in RMSE, which can be attributed to the introduction of outdated information that introduces noise into the current market state prediction. In terms of the attention mechanism, increasing the number of heads h from 4 to 8 yields a significant improvement in representational capacity, while the gains from 8 to 16 heads are marginal and come at a higher computational and memory cost, justifying the selection of h = 8 as the optimal balance for practical deployment. These findings suggest that the FMST framework is robust to hyperparameter variations, confirming its suitability for modeling complex market systems.

### 4.8. Evaluation of Processing Efficiency and System Latency

The operational feasibility of the FMST framework in real-world trading environments is evaluated through a comprehensive end-to-end latency analysis. Given the 1 min temporal resolution utilized for price sensing, it is critical that the multi-stage pipeline—encompassing data acquisition, multimodal encoding, and model inference—completes within this threshold to facilitate real-time decision-making. The hardware platform, featuring a high-performance GPU and multi-core CPU, provides the necessary computational throughput for these high-frequency operations.

Quantitative measurements indicate that the automated retrieval and temporal synchronization of features across the four modalities require an average of 180 ms per sampling interval. The semantic encoding of financial news and announcements, which represents the most computationally demanding stage, is executed in approximately 450 ms using the GPU-accelerated environment. The final model inference stage, responsible for cross-modal fusion and target prediction, requires only 25 ms. Consequently, the total cumulative latency for a single prediction cycle is approximately 655 ms.

This processing time represents only 1.09% of the 60,000-ms (1 min) trading window, providing a substantial buffer that allows the system to respond nearly instantaneously to incoming market information and information shocks. We further discuss the implications of this latency on the ability to exploit market opportunities. In competitive trading environments, the window for capturing alpha from news and announcements is often narrow. However, because the FMST framework maintains a sub-second end-to-end delay, the information advantage gained from news sentiment is preserved for the vast majority of the 1 min trading interval. While ultra-high-frequency strategies operating at the microsecond level might be constrained by semantic encoding times, our analysis suggests that for the targeted 1 min resolution, the system effectively captures and acts upon market opportunities before they are fully absorbed into the price. These results confirm that the FMST framework effectively handles the high-frequency data streams generated by modern trading systems without encountering computational drift or I/O bottlenecks.

### 4.9. Discussion

The proposed FMST framework provides a new perspective for data analysis in complex market systems from the viewpoint of multi-source information sensing and distributed collaborative modeling. In real-world economic systems, market states are typically driven by multiple information sources, such as trading behaviors, capital flows, news events, and macroeconomic changes. These information sources are usually distributed across different institutions and platforms, forming a typical distributed information structure. For example, in financial markets, exchanges record high-frequency price and order book changes, news agencies publish corporate announcements and policy information, and macroeconomic institutions release indicators such as interest rates and inflation levels. These heterogeneous information sources jointly constitute key signals reflecting market dynamics. By modeling these heterogeneous data sources as multimodal market-sensor signals, the proposed framework enables the model to understand market state variations from a holistic perspective, thereby improving predictive performance. Experimental results demonstrate that multimodal information fusion significantly enhances the ability of the model to perceive dynamic market changes, indicating that market behavior is not determined solely by price sequences but rather emerges from the joint influence of multiple information sources.

The superior performance of the FMST framework can be theoretically attributed to its capacity for adaptive information filtering and noise suppression in highly non-linear environments. Unlike traditional models that treat all inputs with equal structural importance, the mixture-of-experts mechanism and attention-based gating effectively act as a dynamic regime-classifier. From the perspective of behavioral finance and market microstructure, information value is not constant; it is regime-dependent. During periods of liquidity stress, order book depth carries more weight, whereas during policy shifts, macroeconomic and news sensors become dominant. By allowing the model to switch between specialized experts, the FMST framework approximates the complex decision-making processes of sophisticated market participants who adjust their strategies based on the prevailing information regime. This architectural choice explains why the model maintains higher robustness across different market conditions compared to static fusion approaches.

An important consideration in this multi-source modeling approach is the anticipation effect of market participants regarding macroeconomic announcements. It is widely recognized in the financial literature that official indicators may be partially or fully incorporated into asset prices before their formal release as participants adjust their positions based on expectations. In the FMST framework, this phenomenon is addressed through the fusion of high-frequency transactional data and real-time news sentiment. While macroeconomic indicators provide the long-term baseline, the high-frequency price and order book sensors capture the immediate market reactions and speculative behaviors that precede official data releases. By monitoring shifts in sentiment and order flow intensity in the lead-up to scheduled announcements, the model can effectively characterize how market expectations are being priced in. This interaction between low-frequency economic context and high-frequency behavioral signals allows for a more comprehensive representation of market efficiency and the information transmission process.

However, from a practical and real-world perspective, it must be acknowledged that a reduction in statistical prediction errors does not necessarily translate into a profitable trading strategy. While the FMST framework demonstrates superior performance in terms of root mean square error and DA, the transition from predictive signals to abnormal returns involves complex factors such as transaction costs, market impact, and liquidity constraints. Furthermore, the impact of prediction errors is often asymmetric in financial markets; for instance, the financial loss incurred from a false positive signal during a market downturn may be significantly larger than the gain from a correct prediction during stable periods. Consequently, correctly predicting market direction is only one component of a successful trading system. The proposed framework should be viewed as a high-fidelity information sensing layer that provides improved inputs for decision-making. In actual deployment, it would require integration with robust risk management modules and execution strategies that account for the non-linear utility of gains and losses.

In practical applications, the proposed framework exhibits substantial potential for resolving information silos and mitigating information asymmetry. In cross-market risk monitoring, complete transaction data are often not directly shared among exchanges in different regions, yet regulatory institutions still need to identify potential systemic risks. Through federated collaborative learning, different market nodes can jointly train predictive models without sharing raw data, thereby producing more stable risk prediction systems. In asset management and quantitative investment, multimodal fusion models can simultaneously utilize market transaction data and textual information such as corporate announcements, macroeconomic policy changes, and international news events, enabling more comprehensive evaluation of market trend variations. Furthermore, in cross-regional financial market analysis, different markets often exhibit diverse trading structures and investor behaviors. Federated learning mechanisms allow knowledge from different regional markets to be integrated, enabling the model to learn more universal market patterns and thereby improving cross-market prediction capability. Overall, the FMST framework demonstrates the potential of combining multimodal sensing with federated collaborative learning for modeling complex economic systems and provides a practical technical pathway for financial risk monitoring, market trend analysis, and cross-regional economic data collaboration [23].

### 4.10. Limitations and Future Work

Although the proposed FMST framework achieves promising results in multimodal market data modeling and distributed collaborative learning, several issues remain to be further explored. The constructed multimodal market-sensor dataset mainly includes price sequences, order book structures, textual information, and macroeconomic indicators. Although these data sources provide relatively comprehensive descriptions of market dynamics, additional information sources exist in real-world economic systems, such as capital flow structures, institutional trading behaviors, and cross-market capital transmission patterns. These factors have not yet been fully incorporated into the current modeling framework. Furthermore, although federated learning enables collaborative modeling without sharing raw data, practical deployment may still face challenges such as communication efficiency, heterogeneous computing capabilities among nodes, and uneven data quality across institutions. These factors may influence training efficiency and model stability. In addition, institutional environments, investor structures, and policy mechanisms may differ substantially across regional markets, and such structural heterogeneity still poses challenges for cross-region prediction tasks.

Beyond these operational constraints, the proposed method also inherently faces conceptual limitations typical of high-dimensional forecasting systems. One such limitation is endogeneity, as the complex feedback loops between news sentiment, macroeconomic expectations, and price movements can lead to simultaneity bias, which deep sequential models may not completely isolate. Furthermore, the use of diverse market sensors can introduce multicollinearity, as signals from the order book and price-volume sequences often contain redundant information, potentially complicating the learning of unique feature contributions. Finally, despite the superior performance of the Transformer architecture, it lacks the explicit interpretability of traditional econometric models like ARIMA [65], making it difficult to perform rigorous causal inference on the predicted outcomes. Future research may extend multimodal data sources and integrate more efficient distributed optimization strategies to further improve the adaptability of the framework in complex economic systems. Moreover, incorporating finer-grained market behavior features and cross-market information propagation mechanisms may further enhance the ability of the model to capture complex market dynamics and provide more reliable support for economic system analysis and risk monitoring.

## 5. Conclusions

Under the background of highly digitalized and information-intensive global trading systems, financial markets continuously generate large volumes of heterogeneous data originating from multiple sources. However, due to the complexity of data types, significant distribution differences, and privacy protection constraints, the development of intelligent analytical frameworks capable of integrating multimodal data and supporting distributed collaborative learning is of great importance for improving prediction capability and risk identification in complex market systems. To address this problem, the main contributions of this study are reflected in three aspects. First, a multimodal market-sensor representation method is proposed, in which price sequences, order book structures, textual information, and macroeconomic indicators are unified and modeled as multimodal time series, thereby constructing a unified data representation capable of reflecting complex market dynamics. Second, a cross-modal fusion model based on the Transformer architecture is designed, in which the attention mechanism enables dynamic interaction and feature fusion among different information sources, thereby improving the model’s capability to characterize complex market structures. Third, a federated collaborative learning mechanism is introduced, allowing data nodes from different market regions or institutions to conduct collaborative model training without sharing raw data, thus enabling cross-region knowledge integration and improving model generalization ability. Extensive experimental results verify the effectiveness of the proposed method. In the main prediction experiment, FMST achieves the best predictive performance on the multimodal market-sensor dataset, where RMSE reaches 0.1136, MAE is 0.0832, and $R^{2}$ increases to 0.8517, while the trend prediction accuracy reaches 74.56%, outperforming baseline models including ARIMA, LSTM, Temporal CNN, Transformer, and FedAvg-LSTM. In the cross-region generalization experiment, FMST also demonstrates stronger stability, with RMSE of 0.1242, $R^{2}$ of 0.8261, and direction prediction accuracy of 72.48%, indicating that the model can effectively adapt to distribution changes across different market environments. The ablation study further demonstrates that the multimodal market-sensor representation module, the cross-modal Transformer fusion module, and the federated collaborative learning module all contribute significantly to the overall performance. Furthermore, in the experiment investigating the number of federated nodes, as the number of participating collaborative nodes increases from a single node to 8 nodes, the prediction error consistently decreases, while direction prediction accuracy improves from 69.21% to 74.56%. These results indicate that the federated collaborative mechanism can effectively integrate multisource market information and significantly enhance model performance.

## Figures and Tables

**Table 1:** Comparison of the proposed FMST framework with existing federated and multimodal methods.

Feature	FedAvg-LSTM	Multimodal-CNN	HA-Fedformer	FMST (Ours)
Data Modality	Single/Static	Multimodal	Multimodal	Dynamic Market-Sensors
Fusion Strategy	Parameter Avg	Concatenation	Knowledge Distill	Cross-modal Transformer
Heterogeneity	Limited	None	Moderate	Adversarial Alignment
Temporal Scale	Uniform	Fixed	Fixed	Multi-scale (MoE)
Privacy Level	High	None	High	High + Distillation

**Table 2:** Statistics and typology of the multimodal market-sensor dataset.

Data Type	Typology	Source	Time Resolution	Data Volume
Price and trading	Numerical (Structured)	NYSE, NASDAQ, SSE, SZSE	1 min	$4.8\times 10^{8}$ records
Order book depth	Numerical (Structured)	Exchange Level-2 APIs	10 s	$1.6\times 10^{8}$ snapshots
Financial news	Text (Unstructured)	Refinitiv, Bloomberg	Event-driven	$1.02\times 10^{6}$ articles
Chinese news	Text (Unstructured)	CNINFO	Event-driven	$7.8\times 10^{5}$ articles
Macro indicators	Numerical (Structured)	World Bank, FRED, NBS	Weekly/Monthly	$2.4\times 10^{4}$ records

**Table 3:** Summary of input data modalities used by different methods.

Method	Input Data Modalities
ARIMA	Univariate historical price sequences
LSTM	Multimodal time-series (Price, Order Book, News, Macro)
Temporal CNN	Multimodal time-series (Price, Order Book, News, Macro)
Transformer	Multimodal time-series (Price, Order Book, News, Macro)
FedAvg-LSTM	Multimodal time-series (Price, Order Book, News, Macro)
FMST (Ours)	Multimodal time-series (Price, Order Book, News, Macro)

**Table 4:** Main prediction performance comparison of different methods on the multimodal market-sensor dataset (Mean ± SD). ↓ indicates that lower values are better, whereas ↑ indicates that higher values are better. * indicates statistically significant improvement compared with the best baseline (p<0.05).

Method	RMSE ↓	MAE ↓	$R^{2}$↑	DA (%) ↑
ARIMA	0.1482 ± 0.0042	0.1127 ± 0.0035	0.7415 ± 0.0152	61.38 ± 1.12
LSTM	0.1316 ± 0.0031	0.0974 ± 0.0028	0.7928 ± 0.0118	66.91 ± 0.95
Temporal CNN	0.1279 ± 0.0029	0.0941 ± 0.0026	0.8046 ± 0.0105	68.27 ± 0.88
Transformer	0.1213 ± 0.0025	0.0895 ± 0.0022	0.8269 ± 0.0094	70.84 ± 0.82
FedAvg-LSTM	0.1248 ± 0.0028	0.0917 ± 0.0025	0.8172 ± 0.0102	69.93 ± 0.91
FMST (Ours)	0.1136 ± 0.0021 *	0.0832 ± 0.0019 *	0.8517 ± 0.0081 *	74.56 ± 0.75 *

**Table 5:** Cross-region generalization performance comparison under train-test transfer across different market regions (Mean ± SD). ↓ indicates that lower values are better, whereas ↑ indicates that higher values are better. * indicates statistically significant improvement compared with the best baseline (p<0.05).

Method	RMSE ↓	MAE ↓	$R^{2}$↑	DA (%) ↑
ARIMA	0.1629 ± 0.0051	0.1246 ± 0.0043	0.7028 ± 0.0185	58.94 ± 1.34
LSTM	0.1475 ± 0.0038	0.1093 ± 0.0032	0.7516 ± 0.0142	63.17 ± 1.15
Temporal CNN	0.1438 ± 0.0035	0.1058 ± 0.0029	0.7639 ± 0.0131	64.42 ± 1.08
Transformer	0.1361 ± 0.0031	0.0996 ± 0.0027	0.7894 ± 0.0118	67.81 ± 0.98
FedAvg-LSTM	0.1337 ± 0.0029	0.0979 ± 0.0025	0.7972 ± 0.0105	68.56 ± 0.92
FMST (Ours)	0.1242 ± 0.0024 *	0.0908 ± 0.0021 *	0.8261 ± 0.0092 *	72.48 ± 0.86 *

**Table 6:** Ablation study of different components in the proposed FMST framework (Mean ± SD). ↓ indicates that lower values are better, whereas ↑ indicates that higher values are better. * indicates statistically significant improvement compared with the best baseline (p<0.05).

Model Variant	RMSE ↓	MAE ↓	$R^{2}$↑	DA (%) ↑
w/o Multimodal Market-Sensor Representation	0.1267 ± 0.0028	0.0936 ± 0.0025	0.8085 ± 0.0102	68.93 ± 0.91
w/o Cross-Modal Fusion Transformer	0.1239 ± 0.0026	0.0912 ± 0.0024	0.8168 ± 0.0098	70.14 ± 0.88
w/o Federated Collaborative Learning	0.1208 ± 0.0024	0.0887 ± 0.0022	0.8249 ± 0.0091	71.36 ± 0.85
FMST (Full Model)	0.1136 ± 0.0021 *	0.0832 ± 0.0019 *	0.8517 ± 0.0081 *	74.56 ± 0.75 *

**Table 7:** Performance comparison with different combinations of input data modalities (Mean ± SD). ↓ indicates that lower values are better, whereas ↑ indicates that higher values are better. * indicates statistically significant improvement compared with the best baseline (p<0.05).

Data Configuration	RMSE ↓	MAE ↓	$R^{2}$↑	DA (%) ↑
Price and Trading only	0.1284 ± 0.0029	0.0948 ± 0.0026	0.8097 ± 0.0105	69.21 ± 0.92
+Order Book	0.1221 ± 0.0025	0.0898 ± 0.0022	0.8214 ± 0.0094	71.05 ± 0.84
+News and Announcements	0.1172 ± 0.0023	0.0861 ± 0.0020	0.8385 ± 0.0088	73.18 ± 0.79
Full (All Modalities)	0.1136 ± 0.0021 *	0.0832 ± 0.0019 *	0.8517 ± 0.0081 *	74.56 ± 0.75 *

**Table 8:** Performance comparison under different numbers of federated clients (Mean ± SD). ↓ indicates that lower values are better, whereas ↑ indicates that higher values are better. * indicates statistically significant improvement compared with the best baseline (p<0.05).

Number of Clients	RMSE ↓	MAE ↓	$R^{2}$↑	DA (%) ↑
Local Training (Single Node)	0.1284 ± 0.0029	0.0948 ± 0.0026	0.8097 ± 0.0105	69.21 ± 0.92
2 Clients	0.1237 ± 0.0026	0.0913 ± 0.0024	0.8206 ± 0.0098	70.54 ± 0.88
4 Clients	0.1196 ± 0.0023	0.0879 ± 0.0021	0.8338 ± 0.0089	71.82 ± 0.82
6 Clients	0.1168 ± 0.0022	0.0856 ± 0.0020	0.8425 ± 0.0084	73.04 ± 0.78
8 Clients	0.1136 ± 0.0021 *	0.0832 ± 0.0019 *	0.8517 ± 0.0081 *	74.56 ± 0.75 *

**Table 9:** Sensitivity analysis results of the FMST framework under different hyperparameter settings (Mean ± SD). ↓ indicates that lower values are better, whereas ↑ indicates that higher values are better. * indicates statistically significant improvement compared with the best baseline (p<0.05).

Parameter	Value	RMSE ↓	$R^{2}$↑	DA (%) ↑
Sliding-window Length (*L*)	15	0.1214 ± 0.0026	0.8256 ± 0.0095	71.04 ± 0.88
	30	0.1136 ± 0.0021 *	0.8517 ± 0.0081 *	74.56 ± 0.75 *
	45	0.1145 ± 0.0023	0.8492 ± 0.0084	74.12 ± 0.79
	60	0.1158 ± 0.0025	0.8451 ± 0.0088	73.65 ± 0.83
Attention Heads (*h*)	4	0.1189 ± 0.0024	0.8365 ± 0.0092	72.41 ± 0.85
	8	0.1136 ± 0.0021 *	0.8517 ± 0.0081 *	74.56 ± 0.75 *
	16	0.1132 ± 0.0020	0.8524 ± 0.0079	74.68 ± 0.72

## Data Availability

To ensure the reproducibility of our research, the source code and comprehensive implementation details of the FMST framework have been prepared and are hosted on GitHub https://github.com/Aurelius-04/FMST.git (accessed on 11 April 2026). We are committed to making this repository publicly accessible immediately upon the formal acceptance of the manuscript. Regarding the multimodal market-sensor dataset, while certain components are subject to privacy constraints and third-party licensing agreements, the processed data and sample subsets are available from the corresponding author upon reasonable request.
